# Molecular epidemiological studies on animal trypanosomiases in Ghana

**DOI:** 10.1186/1756-3305-5-217

**Published:** 2012-10-01

**Authors:** Jesca Nakayima, Ryo Nakao, Andy Alhassan, Charles Mahama, Kofi Afakye, Chihiro Sugimoto

**Affiliations:** 1 Division of Collaboration and Education, Research Center for Zoonosis Control, Hokkaido University, Kita 20, Nishi 10, Kita-ku, Sapporo, Hokkaido, 001-0020, Japan; 2 National Livestock Resources Research Institute (NaLIRRI), P.O Box 96, Tororo, Uganda; 3 Veterinary Services Department, P.O Box M161, Accra, Ghana

**Keywords:** Trypanosomiasis, Human African Trypanosomiasis, Ghana, PCR

## Abstract

**Background:**

African trypanosomes are extracellular protozoan parasites that are transmitted between mammalian hosts by the bite of an infected tsetse fly. Human African Trypanosomiasis (HAT) or sleeping sickness is caused by *Trypanosoma brucei rhodesiense* or *T. brucei gambiense,* while African Animal Trypanosomiasis (AAT) is caused mainly by *T. vivax*, *T. congolense, T. simiae,T. evansi* and *T. brucei brucei*. Trypanosomiasis is of public health importance in humans and is also the major constraint for livestock productivity in sub-Saharan African countries. Scanty information exists about the trypanosomiasis status in Ghana especially regarding molecular epidemiology. Therefore, this study intended to apply molecular tools to identify and characterize trypanosomes in Ghana.

**Methods:**

A total of 219 tsetse flies, 248 pigs and 146 cattle blood samples were collected from Adidome and Koforidua regions in Ghana in 2010. Initial PCR assays were conducted using the internal transcribed spacer one (ITS1) of ribosomal DNA (rDNA) primers, which can detect most of the pathogenic trypanosome species and *T. vivax-*specific cathepsin L-like gene primers. In addition, species- or subgroup-specific PCRs were performed for *T. b. rhodesiense*, *T. b. gambiense*, *T. evansi* and three subgroups of *T. congolense*.

**Results:**

The overall prevalence of trypanosomes were 17.4% (38/219), 57.5% (84/146) and 28.6% (71/248) in tsetse flies, cattle and pigs, respectively. *T. congolense* subgroup-specific PCR revealed that *T. congolense* Savannah (52.6%) and *T. congolense* Forest (66.0%) were the endemic subgroups in Ghana with 18.6% being mixed infections. *T. evansi* was detected in a single tsetse fly. Human infective trypanosomes were not detected in the tested samples.

**Conclusion:**

Our results showed that there is a high prevalence of parasites in both tsetse flies and livestock in the study areas in Ghana. This enhances the need to strengthen control policies and institute measures that help prevent the spread of the parasites.

## Background

African trypanosomes are extracellular protozoan parasites that are transmitted between mammalian hosts by the bite of an infected tsetse fly. Human African Trypanosomiasis (HAT) or sleeping sickness is caused by *Trypanosoma brucei rhodesiense* or *T. brucei gambiense.* The two subspecies are geographically distinct; the separation can be approximated to *T. b. gambiense* present west of the Great Rift Valley and *T. b. rhodesiense* to the east [1]. Livestock is a major reservoir of HAT caused by *T. b. rhodesiense*[2]. Trypanosomiasis in livestock has a significant impact on agricultural productivity and is caused mainly by *T. congolense*, *T. vivax*, *T. simiae*, *T. evansi* and *T. brucei brucei*[3]. *T. evansi*, which is most closely related to *T. b. brucei*, is not transmitted by tsetse flies but mechanically transmitted by biting flies [4].

Scanty information exists about the trypanosomiasis status in Ghana, especially regarding molecular epidemiology. In 2003, a 10-month-old Ghanaian boy recovered from a *T. brucei* infection [5]. The identity of the trypanosome was determined by DNA extraction from the archived stained blood slides followed by sequential application of PCR assays that are specific for the order, subgenus, species and subspecies. The epidemiology of bovine trypanosomosis was investigated in two districts (Savelugu and West Mamprusi) of Northern Ghana with different land use and environmental characteristics [6]. The land use intensity and environmental change was suspected to be higher in the Savelugu district. The parasitological and serological prevalence of bovine trypanosomoses was significantly higher in West Mamprusi (16% and 53%, respectively) than in Savelugu district (8% and 24%, respectively). A cross-sectional entomological survey conducted along the White Volta River and its tributaries confirmed the presence of only *Glossina palpalis gambiensis* and *G. tachinoides*[6].

Prohibitive costs and widespread perception that diagnostic PCR technology is complex slows down its adoption. For instance, in the case of the species-specific diagnosis, five different PCR assays per sample would be required to screen for *T. vivax, T. brucei,T. congolense, T. simiae* and *T. evansi.* The tests would consume considerable time, labour and costs. If the cost constraints are overcome, efforts should be directed towards minimizing sample handling and decreasing the possibility of contamination, at the same time raising the potential to function efficiently in the hands of moderately trained technical staff [7]. The use of the internal transcribed spacer one (ITS1) of ribosomal DNA (rDNA) based primers as a universal diagnostic test for all pathogenic trypanosomes considerably overcomes the above constraints. ITS1 PCR detects eleven pathogenic *Trypanosoma* species in a single PCR, thereby saving time and costs as compared to species-specific PCR. The expected products of ITS1 PCR are species-specific with size differences as indicated: members of subgenus *Trypanozoon* (*T. b. brucei*, *T. evansi*, *T. b. rhodesiense* and *T. b. gambiense*) a constant product of approximately 480 bp; *T. congolense* Savannah subgroup 700 bp, *T*. *congolense* Kilifi subgroup 620 bp, *T. congolense* Forest subgroup 710 bp, *T. simiae* 400 bp, *T. simiae tsavo* 370 bp, *T. godfreyi* 300 bp, and *T. vivax* 250 bp [8-11]. By reducing the number of reactions per sample, the test effectively reduces the cost of PCR and time required for diagnosis.

Previous research on trypanosomiasis in Ghana [6] has employed mainly parasitological and serological tools, which are less accurate. Progress in diagnosis, treatment and epidemiology of trypanosomiasis depends on the existence of specific and sensitive diagnostic tools. Inherent shortcomings of serologic and parasitologic diagnostic methods can be overcome by molecular techniques. Accurate and efficient identification of the trypanosome species present in the fly vectors and vertebrate hosts is vital to assess the disease risk in Ghana.

## Methods

### Study area

**Figure 1:**
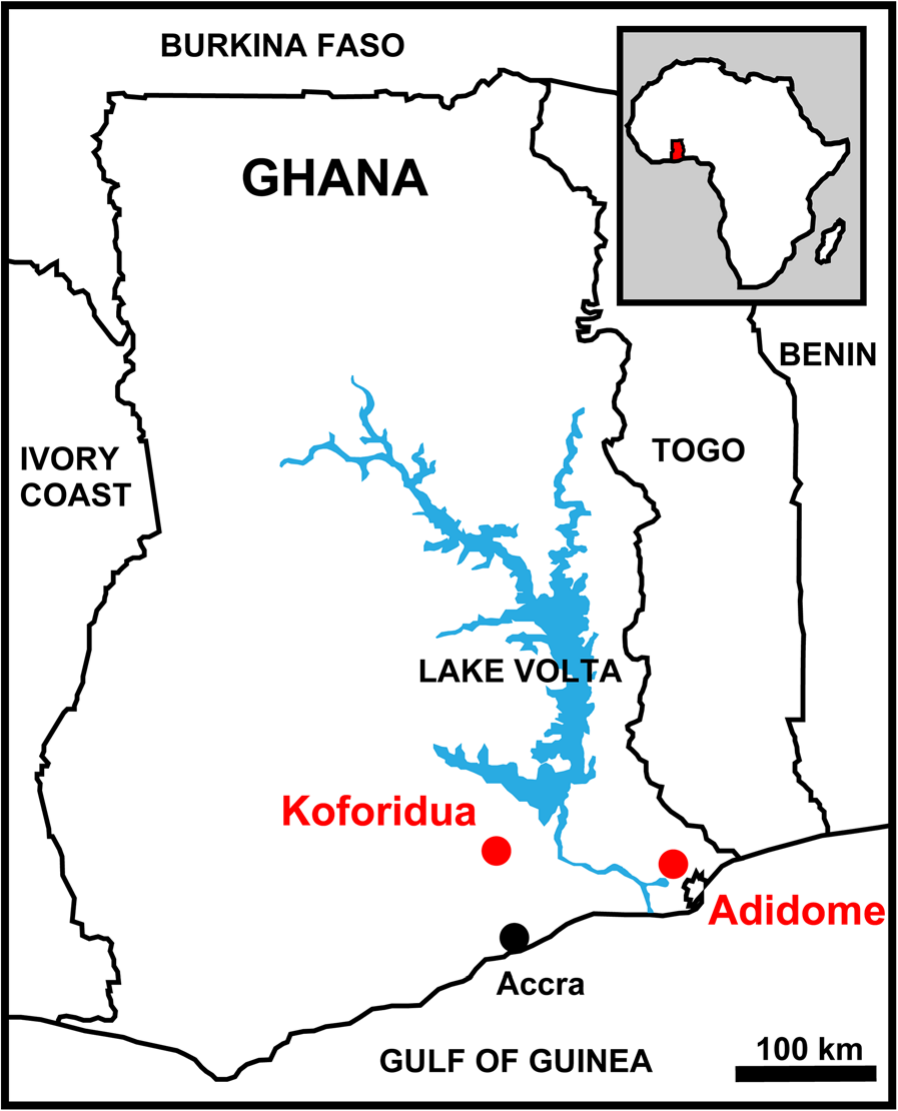
**Map of Ghana showingthe sites where samplingwas conducted.** Tsetse flies, cattle and pig blood were collected from Koforidua and Adidome areas in the south of the country. Sample sites for this study are indicated in red.

The study was conducted on tsetse flies, pig and cattle blood samples collected from Adidome (Latitude: 6° 4' 26.6592" and Longitude: 0° 29' 59.2512") and cattle blood from Koforidua (Latitude: 6° 5' 14.9316" and Longitude: 0° 15' 44.82") areas in Ghana (Figure 1), following an outbreak of trypanosomiasis in livestock in these areas in 2010 (A. Alhassan, personal communication).

### Tsetse flies

Tsetse fly samples were collected from Adidome in Ghana. Typically biconical traps were used to catch the tsetse and each trap was baited with two types of chemical attractant: acetone and 3-*n*-propylphenol 4-methyl-phenol and octenol at a ratio of 1:8:4 [12]. Effort was made to situate the traps under the shade of trees to avoid undue fly mortality and flies were collected every morning. The tsetse flies were sexed and preserved in silica gel until DNA was extracted. A total of 219 tsetse flies were analysed.

### Animal blood

Blood samples were collected from 248 pigs and 108 cattle from Adidome and 38 cattle from Koforidua. Approximately 10 ml of whole blood was withdrawn into a heparinised vaccutainer from the jugular vein. Subsequently 100 μl was applied directly onto Flinders Technology Associates filter paper FTA® Cards (Whatman International Ltd., Abington, Cambridge, UK), which were allowed to dry thoroughly prior to storage at room temperature. Ethical approval was obtained by the Research Centre for Zoonosis Control, Hokkaido University, Japan. The study was conducted adhering to this institution's guidelines for animal husbandry. Verbal informed consent was obtained from each owner of livestock prior to the extraction of blood samples by the field team.

### DNA extraction

Tsetse flies were homogenized by Micro Smash MS-100R (TOMY, Tokyo, Japan) in the presence of stainless steel beads (1.0 mm in diameter) for 2 min at 2,500 rpm, followed by DNA extraction with DNAzol (Invitrogen, Carlsbad, CA). The tsetse homogenate was mixed with 1 ml DNAzol reagent prior to the addition of 100% ethanol. The sample was shaken vigorously and left at room temperature for 5 min followed by two washes with 75% ethanol. DNA was precipitated by centrifugation at 12,000 g for 10 min, solubilised in 200 μl of 8 mM NaOH. The solution was then neutralised by adding 2 μl of 1 M HEPES and was stored at −20°C prior to further processing. DNA extraction from FTA cards by punching 2 mm discs from the sample-saturated cards using an FTA punch and washing three times with distilled water and eluting the DNA in 100 μl of PCR buffer (Promega, Madison, WI).

### PCR

All PCR reactions were conducted using Amplitaq Gold® 360 reagent (Applied Biosystems, Foster City, CA) in a 20 μl reaction volume. The initial PCR screening was performed using ITS1 primers [8] to detect multiple *Trypanosoma* species in a single reaction. However, this primer set was reported to have a low sensitivity against *T. vivax*[8,11,13] presumably because of the low level of sequence similarity between different *T. vivax* isolates. To overcome this problem, this study employed *T. vivax*-specific PCR using the primers TviCatL1 and DTO155 [14]. The samples positive for *Trypanozoon* by ITS1 PCR were further tested by species-specific PCR to characterize *T. b. brucei*, *T. evansi*, *T. b. gambiense* and *T. b. rhodesiense*. When ITS1 PCR generated the PCR products of approximately 700 bp, *T. congolense* subgroup-specific PCR were conducted to distinguish between Savannah, Forest and Kilifi. All the primer sets employed in this study are listed in Table 1. The PCR products were electrophoresed in a 1.5% agarose gel stained with Gel-Red ^TM^ (Biotium, Hayward, CA) and were visualized under UV light.

**Table 1:** PCR primers used in the present study

**Organism**	**Target gene**	**Primer**	**Sequence (5' to 3')**	**Amplicon size (bp)**	**Annealing temperature (°C)**	**Reference**
*Trypanosoma spp.*	ITS1 rDNA	ITS1 CF	CCGGAAGTTCACCGATATTG	Variable	58	[8]
		ITS1 BR	TTGCTGCGTTCTTCAACGAA			
*T. congolense* Kilifi	Satellite DNA monomer	TCK 1	GTG CCC AAA TTT GAA GTG AT	294	55	[15]
		TCK 2	ACT CAA AAT CGT GCA CCT CG			
*T. congolense* Forest	Satellite DNA monomer	TCF 1	GGA CAC GCC AGA AGG TAC TT	350	55	[15]
		TCF 2	GTT CTC GCA CCA AAT CCA AC			
*T. congolense* Savannah	Satellite DNA monomer	TCS 1	CGA GAA CGG GCA CTT TGC GA	316	55	[15]
		TCS 2	GGA CAA AGA AAT CCC GCA CA			
*T. vivax*	Cathepsin L-like gene	DTO 155	TTAAAGCTTCCACGAGTTCTTGATGATCCAGTA	177	65	[14]
		TviCatL1	GCCATCGCCAAGTACCTCGCCGA			
*T. evansi*	RoTat1.2 VSG gene	TeRoTat 920 F	CTGAAG AGGTTGGAAATGGAGAAG	151	58	[16]
		TeRoTat 1070R	GTTTCGGTGGTTCTGTTGTTG TTA			
*T. b. rhodesiense*	SRA gene	Forward	ATAGTGACAAGATGCGTACTCAACGC	284	68	[17]
		Reverse	AATGTGTTCGAGTACTTCGGTCACGCT			
*T. b. gambiense*	TgsGP gene	sense	GCTGCTGTGTTCGGAGAGC	308	63	[18]
		anti-sense	GCCATCGTGCTTGCCGCTC			

### Sequencing analysis

The amplified products of ITS1 PCR were randomly selected (3–5 samples per each amplicon size) and subjected to direct sequencing. The products were treated with ExoSAP-IT (USB Corporation, Cleveland, OH) and sequenced using the BigDye Terminator version 3.1 Cycle Sequencing Kit (Applied Biosystems) and an ABI Prism 3130x genetic analyzer (Applied Biosystems) according to the manufacturer's instructions. The DNA sequences obtained were submitted to the DNA Data Bank of Japan (DDBJ) (http://www.ddbj.nig.ac.jp) under accession nos AB742529 to AB742533.

## Results

### ITS1 &*T. vivax*-specific PCR

**Figure 2:**
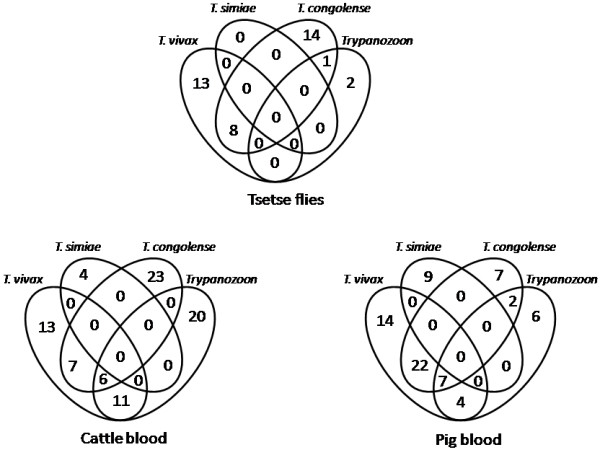
**Status of mixed infectionsbetween different *Trypanosoma *spp .***Trypanozoon *, *T. congolense* and *T. simiae* were screened using ITS1-PCR while *T. vivax* was screened using *T. vivax*-specific cathepsin L-like PCR.

A total of 613 samples including 219 tsetse flies, 146 cattle and 248 pigs blood samples were subjected to ITS1 PCR screening for different trypanosome species and the prevalence of the parasites are indicated in Table 2. Despite that ITS1 PCR detected only 36 positives for *T. vivax* (data not shown), *T. vivax-*specific PCR using cathepsin L-like primers detected 21, 37 and 47 positives in tsetse flies, cattle and pigs, respectively. The overall prevalence of trypanosomes were 17.4% (38/219), 57.5% (84/146) and 28.6% (71/248) in tsetse flies, cattle and pigs, respectively. The trypanosome prevalence was lower in the tsetse flies than in the vertebrate hosts. The predominant species in vertebrate hosts was *T. vivax*, while in tsetse flies *T. congolense* was detected as a predominant species followed by *T. vivax*. In all samples *T. simiae* was the least common. Figure 2 indicates the frequencies of mixed infection in each sample. The mixed infections with two different *Trypanosoma* species were observed in 9, 18 and 28 individuals of tsetse flies, cattle and pigs, respectively. Three different parasite species were detected in 6 cattle and 7 pig blood samples.

**Table 2:** Overall prevalence of trypanosomes

**Sample type**	**Total no. of samples tested**	**ITS1 PCR**			***T. vivax-*specific PCR**	**No. of positives**
		***T. congolense***	***T. simiae***	***Trypanozoon***		
Tsetse flies	219	23	0	3	21	47
Cattle	146	36	4	37	37	114
Pigs	248	38	9	19	47	113
Total	613	97	13	59	105	

### ITS1 sequencing analysis

A total of five different sequences (249, 387, 470, 475 and 725 bp in size) were recovered by sequencing analysis of ITS1 PCR products. These sequences were compared with those available in public databases by using nucleotide BLAST at NCBI website (http://blast.ncbi.nlm.nih.gov/Blast.cgi). The sequences of the 249- and 475-bp products were 100% identical to *T. vivax* (GenBank accession number: HE573019) and *T. evansi* (AY912278), respectively. The sequences of the 387-, 470- and 725-bp products showed 99%, 98% and 96% identities with *T. simiae* (AB625446), *T. brucei* (AC159414) and *T. congolense* (TCU22319), respectively.

### PCR for human infective trypanosomes

The samples positive for *Trypanozoon* by ITS1 PCR were subjected to PCR assays specific for *T. b. rhodesiense* and *T. b. gambiense*. All the reactions were negative and human infective trypanosomes were not detected in the tested samples.

### *T. evansi*-specific PCR

Having detected *T. evansi* in a tsetse fly by sequence analysis of ITS1 PCR product, we conducted *T. evansi*-specific PCR using RoTat 1.2 VSG gene primers for the samples positive for *Trypanozoon* by ITS1 PCR. However, all the samples including one positive for *T. evansi* by sequencing analysis, were negative.

### *T. congolense* subgroup-specific PCR

The samples positive for *T. congolense* by ITS1 PCR were subjected to *T. congolense* subgroup-specific PCRs to ascertain the subgroups endemic in Ghana. Out of 97 samples, 51 and 64 samples were tested positive for subgroups Savannah and Forest, respectively (Table 3). The Kilifi subgroup was not detected in the tested samples. The Forest subgroup was predominant in tsetse flies and pigs, while the Savannah subgroup was predominant in cattle. Mixed infections between subgroups Savannah and Forest were detected in 3 cattle and 15 pig samples.

**Table 3:** ***T. congolense* subgroup-specific PCR results **

**Sample type**	**Total no. of samples tested**	**Savannah**	**Forest**	**Kilifi**	**Mixed infection**
Tsetse flies	23	0	23	0	0
Cattle	36	36	3	0	3
Pigs	38	15	38	0	15
Total	97	51	64	0	18

## Discussion

There are 11 different pathogenic trypanosomes known to exist in Africa. The primers ITS1 CF and ITS1 BR, previously designed to amplify the ITS1 region of all pathogenic trypanosome species, give PCR products with species-specific sizes and thus enable multiplex detection of different parasite species [8]. This assay system was successfully applied to identify and characterize trypanosomes in Ghana with some limitations discussed below.

A total of 613 samples were screened for trypanosome infections by ITS1 PCR, of which 59 samples tested positive for *Trypanozoon*. These were further tested for the presence of human infective trypanosomes, however, none of them were positive. Since the livestock and wild animals are known to act as reservoirs for HAT [19-21], it is important to continue active surveillance in animals to understand the transmission cycle of HAT in endemic areas.

*T. evansi* was detected in a single tsetse fly sample despite being classified as non tsetse- transmitted trypanosomes (NTTT) [4]. This parasite is known to be mechanically transmitted by biting flies such as the genera *Tabanus and Stomoxys*, enabling a world-wide distribution even outside the tsetse belt of Africa. Thus, the detection of *T. evansi* in a tsetse fly might reflect the existence of carrier animals in the vicinity of tsetse flies. Since the ITS1 PCR product size of *T. evansi* is similar to one of *T. brucei*, sequencing analysis was key to differentiating between the two infections. Furthermore, *T. evansi*-specific PCR based on RoTat 1.2 VSG gene failed in this study and thus might not be reliable in epidemiological surveys and diagnosis of *T. evansi* as reported elsewhere [22]. These problems make it difficult to assess the possibility of *T. evansi* endemicity in Ghana.

*T. congolense* subgroup-specific PCR did not detect Kilifi in Ghana (Table 3). This result seems natural considering that it is thought to be an East African subgroup first isolated from livestock in 1982 on a ranch at Kilifi on the Kenyan coast [23]. Both Savannah and Forest were the endemic subgroups in Ghana and mixed infections between the two subgroups were recorded at 18.6%. The Forest subgroup could be the most prevalent in Ghana possibly because of the humid equatorial forest ecosystems of West Africa. Considering that the Savannah subgroup was reported to be more virulent than the Forest subgroup [24], a high prevalence of the Savannah subgroup in cattle may indicate that the parasites were introduced recently into the tested herds.

The high prevalence of *T. vivax* corroborates other findings in domestic animals [25] as well as wild animals [20,26,27]. This may result from the level of pathogenicity of this trypanosome, which is generally low and better controlled by animals [28], and/or from the mechanical transmission, which has been reported in *T. vivax, T. evansi* and to a certain extent *T. congolense*[29]*.* The lower prevalence of *T. congolense* with respect to *T. vivax* in animals may result from higher parasitemia in *T. congolense* infections, accompanied by serious anaemia, which leads to the rapid death of the host animal [30,31]. The very low prevalence of *T. simiae* as previously reported in pigs [19,32] and wild animals [26,27] may indicate a low transmission of the parasite in the studied localities of Ghana and is also likely due to its high pathogenicity because pigs infected with this trypanosome species would not probably survive the acute, severe and fatal nature of this parasite.

In large-scale epidemiological studies, FTA cards are becoming increasingly popular for the rapid collection and archiving of a large number of samples. However, there are some difficulties in the downstream processing of these cards, which is essential for the accurate diagnosis of infection. It should be noted that the use of FTA cards for sample preparation has some limitations. The main one is that at low trypanosome intensities (i.e. animals with a very low level infection and chronic infections) there is an uneven distribution of parasite DNA on the cards and that some positive cards can give a negative result because the wrong part of the card was sampled [33,34]. This has an implication that the results presented from the FTA cards may actually be underestimates of the true prevalence. Therefore, in order to decrease the probability of false negative results from using a single disc, examination of more discs would give more accurate estimation of the disease prevalence [33,34].

The mixed infections with two or three different *Trypanosoma* species were commonly observed in both cattle and pigs (Figure 2). These co-infections have also been documented previously with multiple trypanosome species [35,36]. This molecular epidemiological work confirmed the abundance of mixed infections in the field, which could not have been detected by the classical parasitological methods. Mixed infections could be a result of high chances of trypanosome infections by tsetse flies and/or a case of chronic infection in susceptible hosts. This points towards the severity of AAT, exacerbating animal losses incurred by farmers such that deliberate efforts need to be in place to control tsetse flies in order to break the transmission cycle to domestic animals and thus improve livestock production and productivity in the endemic area. AAT risk is usually linked to the density of the vector and the trypanosome infection rates [36].

## Conclusion

*Trypanosoma* parasites circulate within Ghana at high prevalence in vertebrate hosts and tsetse flies. *T. brucei* was highly prevalent in the study areas but no human infective trypanosomes were detected. In livestock, the endemic *T. congolense* subgroups were Savannah and Forest while Kilifi was not detected. *T. simiae* was detected only in pig and cattle samples. PCR results indicated high prevalence of *T. vivax* in both tsetse flies and vertebrate hosts. *T. evansi* was detected in a tsetse fly sample. Our results provide insight into the epidemiology of trypanosomiasis in Ghana based on highly sensitive and specific molecular techniques. Intervention to control the disease by the various stakeholders is highly recommended.

## Competing interests

The authors declare that they have no competing interests.

## Authors’ contributions

JN performed PCR and sequencing, conducted data analysis and drafted the manuscript. RN guided and directed the study, AA collected the samples, CM & KA participated in sample collection and CS supervised the study. All authors read and approved the final manuscript.
